# Supplementary material for: Effects of Gonadotropin-Releasing Hormone (GnRH) and Its Analogues on the Physiological Behaviors and Hormone Content of Tetrahymena pyriformis
Source: Int J Mol Sci. 2019 Nov 14;20(22):5711. doi: 10.3390/ijms20225711 (PMC6888530; doi:10.3390/ijms20225711)
Supplement: Supplementary file 1 [file ijms-20-05711-s001.pdf]

International Journal of *Molecular Sciences*

MDPI

Supplementary Material

Effects of gonadotropin-releasing hormone (GnRH) and its analogues on the physiological behaviors and hormone content of *Tetrahymena pyriformis*.

Eszter Lajkó [1], Éva Pállinger[1], Zsombor Kovács[1], Ildikó Szabó[2] and László Kőhidai [1,*]

[1] Department Genetics, Cell- and Immunobiology, Semmelweis University, Nagyvárad tér 4., Budapest, 1089, Hungary; lajesz@gmail.com (E.L.), pallinger.eva@med.semmelweis-univ.hu (É.P.), zsomborkovacs@freemail.hu (Zs.K.), kohlasz2@gmail.com (L.K.)

[2] Research Group of Peptide Chemistry, Hungarian Academy of Sciences, Eötvös Loránd University, Pázmány Péter sétány 1/A, Budapest, 1117, Hungary; szaboi8@gmail.com (I.Sz.)

* Correspondence: kohlasz2@gmail.com; Tel.: +36-1-210-2930/56232

Received: date; Accepted: date; Published: date

Table S1 Chemokinetic effects of GnRH derivatives on *Tetrahymena* – Mean velocity and tortuosity data of the treated and the control cells

Tested GnRH peptides	Chemokinetic effects	
	Mean velocity ± SD [µm/sec]	Tortuosity ± SD
control	562.6 ± 30.0	1.57 ± 0.15
GnRH-I	429.1** ± 28.9	2.81** ± 0.35
GnRH-III	439.6** ± 32.5	2.69** ± 0.35
Ac-SHDWKPG-NH_2	328.9*** ± 29.7	1.90 ± 0.17
[GnRH-III(C)]$_2$	519.5 ± 48.7	2.03 ± 0.25
[GnRH-III(CGFLG)]$_2$	346.2*** ± 33.6	3.12** ± 0.40

The effects of the GnRH peptides were studied in the following concentrations: GnRH-I: 10^{-6} M, GnRH-III: 10^{-6} M, Ac-SHDWKPG-NH_2: 10^{-6} M, [GnRH-III(C)]$_2$: 10^{-11} M, [GnRH-III(CGFLG)]$_2$: 10^{-6} M. Data represent the mean of 4 parallels ± SD. The levels of significance are shown as follows: *: p<0.05; **: p<0.01; ***: p<0.001.

International Journal of *Molecular Sciences*

MDPI

Table S2 Cell proliferation modulator effects of the GnRH derivatives on *Tetrahymena* after 6 h – Viable cell number values of the treated and the control cells

GnRH peptides	Viable cell number [cell/ml] ± SD							
	Control	10^{-12} M	10^{-11} M	10^{-10} M	10^{-9} M	10^{-8} M	10^{-7} M	10^{-6} M
**GnRH-I**	14375 ± 468.7	13597 ± 916	12463* ± 483.8	13586 ± 465.4	13003 ± 417	12658** ± 549	13414 ± 432.2	13943 ± 566.4
**GnRH-III**	16315 ± 495.3	14461 ± 812.5	16060 ± 1431.9	15509 ± 1829.1	15520 ± 1095.2	16027 ± 311.5	16459 ± 624	15466 ± 1128.3
**Ac-SHDWKPG-NH_2**	14688 ± 834	16503 ± 1087	15196 ± 1169	15779 ± 713.9	14872 ± 481.2	17856* ± 624.2	16708 ± 960.6	16762 ± 1226
**[GnRH-III(C)]$_2$**	14688 ± 834	16215 ± 246.1	16618 ± 269	16773 ± 1266.6	16200 ± 1105.1	17410 ± 799.9	17539* ± 287.6	18814** ± 1594.5
**[GnRH-III(CGFLG)]$_2$**	11707 ± 409.5	13090 ± 719.2	13165 ± 1268.8	11318 ± 468.6	12236 ± 92.3	12031 ± 421.2	12776 ± 657.1	14234* ± 666.6

Data represent the mean of 4 parallels ± SD. The levels of significance are shown as follows: *: p<0.05; **: p<0.01.

Table S3 Cell proliferation modulator effects of the GnRH derivatives on *Tetrahymena* after 24 h – Viable cell number values of the treated and the control cells

GnRH peptides	Viable cell number [cell/ml] ± SD							
	**Control**	**10^{-12} M**	**10^{-11} M**	**10^{-10} M**	**10^{-9} M**	**10^{-8} M**	**10^{-7} M**	**10^{-6} M**
**GnRH-I**	38999 ± 1257.9	38232 ± 1258.9	36007 ± 1195.3	38005 ± 1177.7	37051 ± 1126.9	36331 ± 984	35381* ± 993.3	37676 ± 1066.3
**GnRH-III**	36083 ± 1014	38934 ± 1017.2	39355 ± 1834.4	37858 ± 1050	37517 ± 365.5	38208 ± 1433.9	38629 ± 751	39374* ± 416
**Ac-SHDWKPG-NH_2**	57392 ± 630.1	54875 ± 3215.4	52542 ± 1044.5	53082 ± 2594.4	56290 ± 935.7	55016 ± 1301.9	55815 ± 676	54238 ± 922.9
**[GnRH-III(C)]$_2$**	57392 ± 630.1	55577 ± 1556.1	55167 ± 1610.6	55052 ± 1015.7	58752 ± 1360.3	61949 ± 4184.7	54281 ± 852.7	52683 ± 1380.3
**[GnRH-III(CGFLG)]$_2$**	51264 ± 1008.9	47952 ± 381.9	49292 ± 514.5	49205 ± 699.9	50652 ± 867.3	51171 ± 1553.4	50598 ± 2637	49443 ± 1900

Data represent the mean of 4 parallels ± SD. The levels of significance are shown as follows: *: p<0.05

International Journal of *Molecular Sciences*

MDPI

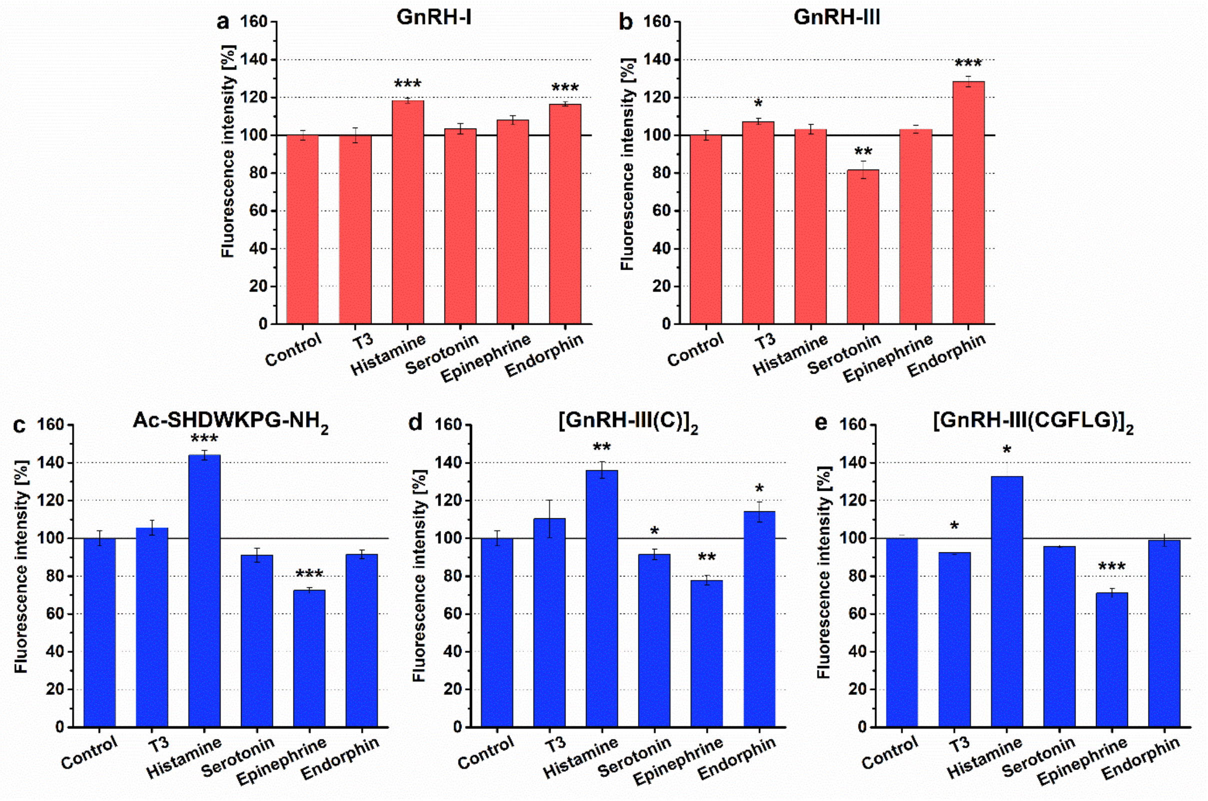

Figure S1 Hormonal effects of GnRH peptides in *Tetrahymena pyriformis* – Numerical changes in the intracellular hormone content normalized to control

The effects of the GnRH peptides were studied in the following concentrations: GnRH-I: 10^{-6} M, GnRH-III: 10^{-6} M, Ac-SHDWKPG-NH_2: 10^{-6} M, $[\text{GnRH-III(C)}]_2$: 10^{-11} M, $[\text{GnRH-III(CGFLG)}]_2$: 10^{-6} M. The mean fluorescence intensity is expressed as a percentage of the untreated control. Data represent the mean of 5 parallels ± SD. The levels of significance are shown as follows: *: $p<0.07$; **: $p<0.01$, ***: $p<0.001$.

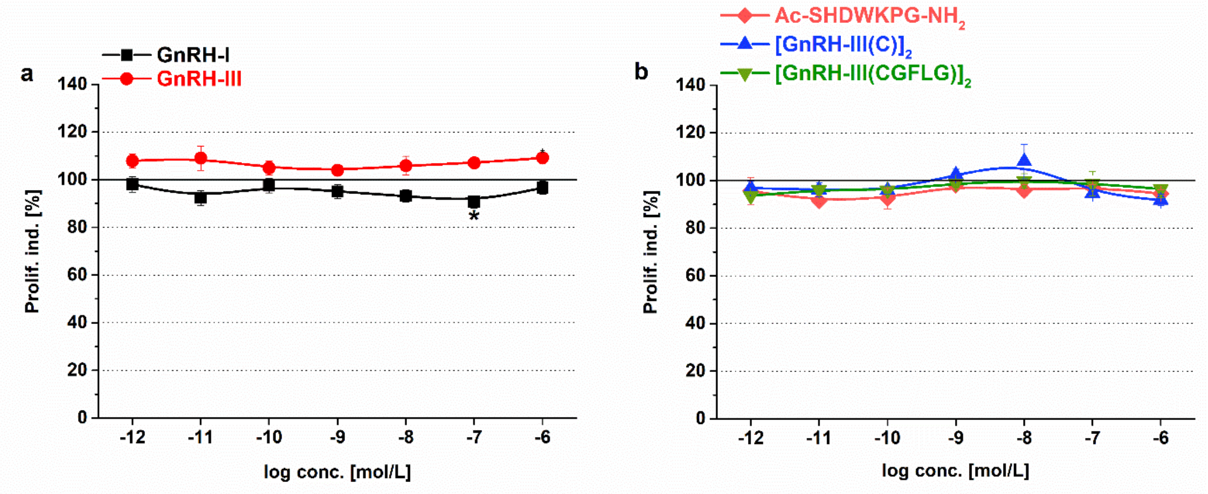

Figure S2 Long term (24 h) effects of the GnRH derivatives on *Tetrahymena* proliferation

Proliferation index (Prolif. ind.) describes the number of viable cells normalized to control. Data represent the mean of 4 parallels ± SD. The level of significance is shown as follows: *: $p<0.05$.
